# Impact of parental and healthcare professional concern on the diagnosis of pediatric sepsis: a diagnostic accuracy study

**DOI:** 10.3389/fped.2023.1140121

**Published:** 2023-04-17

**Authors:** Zoe Sever, Luregn J. Schlapbach, Patricia Gilholm, Melanie Jessup, Natalie Phillips, Shane George, Kristen Gibbons, Amanda Harley

**Affiliations:** ^1^ School of Nursing, Midwifery and Social Work, The University of Queensland, Brisbane, QLD, Australia; ^2^ Child Health Research Centre, University of Queensland, South Brisbane, QLD, Australia; ^3^ Paediatric Intensive Care Unit, Queensland Children’s Hospital, Children's Health Queensland Hospital and Health Service, South Brisbane, QLD, Australia; ^4^ Department of Intensive Care and Neonatology, Children’s Research Center, University Children’s Hospital Zürich, Zürich, Switzerland; ^5^ Emergency Department, Queensland Children’s Hospital, Children's Health Queensland Hospital and Health Service, South Brisbane, QLD, Australia; ^6^ Departments of Emergency Medicine and Children's Critical Care Service, Gold Coast University Hospital, Southport, QLD, Australia; ^7^ Faculty of Health, School of Medicine, and Menzies Health Institute Queensland, Griffith University, Gold Coast, QLD, Australia; ^8^ Critical Care Nursing Management Team, Queensland Children’s Hospital, Brisbane, QLD, Australia

**Keywords:** diagnosis, concern, doctor, nurse, parent, sepsis

## Abstract

**Objective:**

The Surviving Sepsis Campaign recommends systematic screening for sepsis. Although many sepsis screening tools include parent or healthcare professional concern, there remains a lack of evidence to support this practice. We aimed to test the diagnostic accuracy of parent and healthcare professional concern in relation to illness severity, to diagnose sepsis in children.

**Design:**

This prospective multicenter study measured the level of concern for illness severity as perceived by the parent, treating nurse and doctor using a cross-sectional survey. The primary outcome was sepsis, defined as a pSOFA score >0. The unadjusted area under receiver-operating characteristic curves (AUC) and adjusted Odds Ratios (aOR) were calculated.

**Setting:**

Two specialised pediatric Emergency Departments in Queensland

**Patients:**

Children aged 30 days to 18 years old that were evaluated for sepsis

**Intervention:**

None

**Main Results:**

492 children were included in the study, of which 118 (23.9%) had sepsis. Parent concern was not associated with sepsis (AUC 0.53, 95% CI: 0.46–0.61, aOR: 1.18; 0.89–1.58) but was for PICU admission (OR: 1.88, 95% CI: 1.17–3.19) and bacterial infection (aOR: 1.47, 95% CI: 1.14–1.92). Healthcare professional concern was associated with sepsis in both unadjusted and adjusted models (nurses: AUC 0.57, 95% CI-0.50, 0.63, aOR: 1.29, 95% CI: 1.02–1.63; doctors: AUC 0.63, 95% CI: 0.55, 0.70, aOR: 1.61, 95% CI: 1.14–2.19).

**Conclusions:**

While our study does not support the broad use of parent or healthcare professional concern in isolation as a pediatric sepsis screening tool, measures of concern may be valuable as an adjunct in combination with other clinical data to support sepsis recognition.

**Clinical Trial Registration:**

ACTRN12620001340921.

## Introduction

Pediatric Emergency Department (ED) clinicians are challenged by large numbers of children presenting with suspected infection, of which few have, or progress to, sepsis (1, 2). Discriminating sepsis, defined as life-threatening organ dysfunction caused by a dysregulated host response to infection, from other milder infections within the ED remains problematic given that signs and symptoms of sepsis are often vague and non-specific (3, 4). The latest pediatric Surviving Sepsis Campaign (SSC) emphasised the importance of early recognition and prompt management to optimise outcomes for children with sepsis (5). Yet, there is a lack of evidence on optimal tools to assist in the systematic recognition of sepsis in pediatric EDs (6, 7). In addition to physiological markers, patient history, and laboratory tests, there is ongoing debate as to whether the presence of parent or healthcare professional perception for illness severity (“concern”) could improve early recognition of pediatric sepsis, given the role of parents as experts of their child (8). Root-cause-analyses and anecdotal reports on pediatric sepsis fatalities indicate that parents frequently express concerns that the “illness was different” (9). Numerous institutional and national sepsis tools include assessment of parent concern as part of a standard sepsis screening (8). Yet, a systematic review of parent concern identified only one diagnostic accuracy study performed in a community setting which reported that parent concern acted as a “red flag” for clinicians (8, 10).

Despite the apparently obvious rationale to include parent or healthcare professional concern in sepsis screening tools, there is a need for diagnostic accuracy studies. We hypothesised that the presence of parent and/or healthcare professional concern would be associated with a diagnosis of pediatric sepsis. The aim of this study was to determine the diagnostic accuracy of concern for illness severity in the parent, treating nurse and doctor to diagnose sepsis in children evaluated for sepsis in the ED.

## Methods

### Study design

This prospective multicentre observational cohort study utilised a cross-sectional survey (Supplementary Material S2) to assess concern levels for illness severity in the parent, treating nurse, and doctor for children who presented to the ED with suspected infection and were screened *via* the institutional sepsis pathway (11). We specifically included children who were identified as at risk for sepsis according to the institutional sepsis pathway criteria. This pathway is activated based on clinical signs, laboratory findings, and whether parental or healthcare worker concern led to the question “Could this be sepsis?”. Surveys had to be completed at the time closest to triage as part of the assessment during ED, and within no more than 4 h of presentation, in accordance with the National Emergency Access Target (12). Details of the study protocol and analysis plan have been previously published (13). Ethical approval was obtained from the Children's Health Queensland Human Research Ethics Committee (HREC/17/QRCH/85). Completion of the survey by the respondent implied consent to participate in the study. Reporting of this study follows Standards for Reporting of Diagnostic Accuracy (STARD) guidelines (14).

### Participants and setting

Patients aged between 30 days to 18 years old and presenting to two tertiary pediatric EDs (Queensland Children's Hospital, Gold Coast University Hospital, Queensland, Australia) between December 2018 and January 2021 were eligible to participate if they were evaluated for suspected infection through the institutional sepsis pathway and/or undergoing blood culture sampling for suspected infection. Patients whose parents did not speak English or those patients with suspicion of SARS-CoV-2 infection, treated in isolation, were excluded (13). Numbers of children treated for suspected SARS-CoV-2 infection were low in Queensland during the recruitment period.

### Survey tool design and validation

The surveys were designed jointly for the parent, nurse, and doctor as previously reported (13). Raters were asked to specify the degree to which they agreed or disagreed with each of five statements (four for the nurse/doctor), designed to measure concern for illness severity *via* a five-point Likert item (10), with 1 indicating *not concerned* and 5 indicating *extremely concerned*. The surveys did not use the word “sepsis” but rather referred to the perceived “severity of illness (how sick)”. The content validity of the survey was assessed using an exploratory factor analysis and the reliability of the survey was measured through the internal consistency (Cronbach's alpha) and inter-rater reliability (intra-class correlation) (Supplementary Material S3). The four concern questions were found to be valid and reliable measures of the latent construct “concern”.

### Data collection

Information relating to patient demographics, history, physiological measurements, and laboratory results at presentation and during the first 48 h after presentation were collected manually from the medical record. Illness severity was determined using the pediatric Sequential Organ Failure Assessment (pSOFA) score (15, 16). Presence of infection was adjudicated into confirmed bacterial, probable bacterial, confirmed viral, probable viral, and uncertain infection, and into non-infectious causes based on criteria relating to laboratory results and treatment delivery by an independent assessor (Supplementary Material S4) (17). Data was recorded securely in electronic case report forms captured in a purpose-built REDCap study database hosted by The University of Queensland (18, 19).

### Outcomes

The primary outcome was a diagnosis of sepsis, defined as suspected or proven infection (bacterial, viral or both) in the presence of organ dysfunction, operationalized by a pSOFA score >0 at time of assessment in the ED. Sepsis was defined as suspected infection with organ dysfunction to align with the SSC guidelines (5) in the absence of an updated definition for the paediatric sepsis population (20).

Secondary outcomes were defined as (1) suspected or proven infection and development of organ dysfunction (pSOFA score >0) within 48 h of presentation; (2) confirmed or probable bacterial infection independent of organ dysfunction; (3) admission to the pediatric intensive care unit (PICU); and (4) hospital length of stay (LOS). Given controversy surrounding pediatric sepsis definitions (20), sensitivity analyses defining organ dysfunction at presentation and within 48 h of presentation as per the 2005 International Pediatric Sepsis Consensus Conference (IPSCC) were conducted (21, 22).

### Statistical methods

The measure of concern was evaluated in two ways: (1) using the Likert-item measurements of each of the four concern questions, and (2) by using a composite concern score, calculated from the exploratory factor analysis, which was subsequently standardised to have a mean of zero and standard deviation of one. The concern score was compared to the four individual concern questions in relation to the association with the primary outcome, by performing unadjusted logistic regression analyses and comparing the unadjusted odds ratio (OR) and area under the receiver operating characteristic curves (AUC) (Supplementary Material S5). No substantial differences were observed between the composite concern score and each concern question; we therefore reported results on the concern score, and on the best performing question (Question 6: “Indicate how severe you think your child/patient's illness is today”).

For each outcome, regression models were performed for each of the three groups (patients with a parent survey, patients with a nurse survey and patients with a doctor survey) to assess the performance of concern within each group. First, we constructed a baseline model. This model did not include concern and was instead based on physiological data and patient characteristics available upon presentation for children included in the study. Data included, age in months, elevated heart rate and elevated respiratory rate, defined as heart rate/respiratory rate greater than the 90th centile for a child's age (23), irritability and respiratory distress at the time of presentation, and chronic disease (Supplementary Material S6) (7). We then ran an unadjusted model using the composite concern score, and the best performing concern question. Finally, we performed an adjusted model which evaluated the concern score, or the best performing concern question, in addition to the covariates included in the baseline model. Due to the low prevalence of admission to the PICU and sepsis defined by the IPSCC criteria, only unadjusted effects are reported for these outcomes. To evaluate each model, ORs were reported to assess the strength of the association between concern and each outcome, and the AUCs were calculated to assess the predictive performance of each model. For hospital LOS, Cox proportional hazards models were estimated, and the hazard ratio and concordance statistic were reported. Differences in AUC between the unadjusted and adjusted models were calculated using DeLong's method (24). For all analyses, 95% confidence intervals (CIs) are reported in place of *p-*values for all parameters given the exploratory nature of the study. A minimum sample size requirement of 450 participants was determined for a power of 80% to detect a 20% improvement in sensitivity for an outcome prevalence of 10% (25). All analyses were conducted using R statistical software (version.4.1.1) (26).

## Results

### Study cohort overview

**Figure 1:**
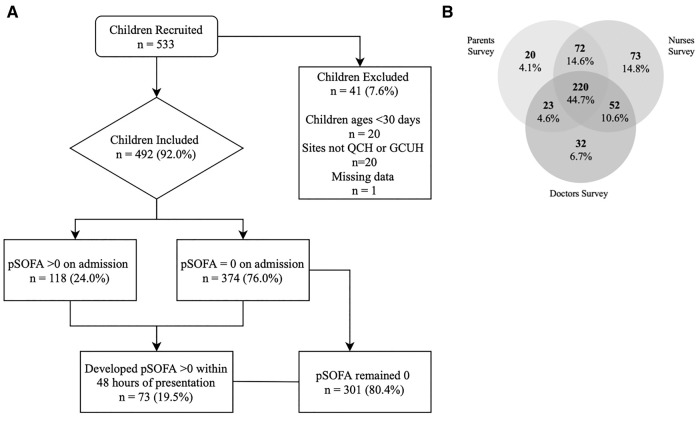
Participant flow diagram (**A**) and venn diagram (**B**) representing the inclusion of patients in relation to sepsis, and the number of surveys completed for parents, nurses and doctors, respectively.

During the study period, 533 patients that were evaluated for suspected sepsis were screened. A total of 492 patients met inclusion criteria, of which 335, 417 and 327 had parent, nurse, and doctor surveys completed, respectively (Table 1, Supplementary Material S7). There were 220 children (44.7%) who had all three surveys completed (Figure 1). The median age of the patients was 26.8 months (IQR 13.2, 70.7) and 268 (54.5%) were male. The median years of experience was seven years for nurses (IQR 4–10; *N* = 338, 81.1% being registered nurses) and six years for doctors (IQR 4–9; *N* = 164, 50.2% being registrars). 58% of parents reported concern for all concern questions, compared to 30% of nurses and 36% of doctors (Supplementary Material S8). In total, 118 children (24.0%) were diagnosed with sepsis at the time of survey completion. 191 (38.8%) children were diagnosed with sepsis within 48 h of presentation. 133 children (27.0%) had confirmed or probable bacterial infection. In total, 31 (6.3%) patients were admitted to PICU and no patients died.

**Table 1:** Baseline characteristics and outcomes are shown for children enrolled the study cohort, according to whether surveys were obtained from parents, nurses, or doctors, respectively. The table shows counts and percentages for categorical variables and median and interquartile ranges for continuous variables. Participants are represented in multiple columns if more than one type of survey (i.e., parents, nurses, or doctors) was obtained. Only one of each survey type per patient was recorded.

Variable	Parent Surveys	Nurse Surveys	Doctor Surveys
	*N* = 335	*N* = 417	*N* = 327
**Demographics**			
Age (months)	31 (14, 79)	22 (13, 61)	28 (14, 80)
Male	183 (55%)	235 (56%)	184 (56%)
**Admission Characteristics**			
Infection symptoms at time of presentation^1^			
Fever	285 (85%)	365 (88%)	278 (85%)
Rash	48 (14%)	51 (12%)	47 (14%)
Altered level of consciousness	40 (12%)	48 (12%)	39 (12%)
Irritability	82 (24%)	102 (24%)	74 (23%)
Seizures	19 (6%)	29 (7%)	24 (7%)
Pain	110 (33%)	119 (29%)	90 (28%)
Nausea/Vomiting	99 (30%)	116 (28%)	91 (28%)
Diarrhoea	23 (7%)	35 (8%)	25 (8%)
Respiratory distress/apnoea	43 (13%)	59 (14%)	58 (18%)
Cough	89 (27%)	121 (29%)	107 (33%)
Pale/cyanotic episode	28 (8%)	38 (9%)	27 (8%)
Cold extremities	7 (2%)	11 (3%)	13 (4%)
Skin/wound infection	11 (3%)	11 (3%)	9 (3%)
Other	85 (25%)	103 (25%)	96 (29%)
**Infection type**			
Definite bacterial infection	58 (17%)	60 (14%)	53 (16%)
Probable bacterial infection	46 (14%)	53 (13%)	42 (13%)
Unknown bacterial or viral infection	62 (19%)	80 (19%)	64 (20%)
Probable viral infection	106 (32%)	148 (35%)	101 (31%)
Definite viral infection	45 (13%)	56 (13%)	52 (16%)
Non-bacterial, non-viral infection or non-infectious illness	18 (5%)	20 (5%)	15 (5%)
**Vital Signs**			
Heart rate >90th centile for age	141 (42%)	208 (50%)	160 (49%)
Respiratory rate >90th centile for age	67 (20%)	93 (22%)	74 (23%)
Chronic Disease	60 (18%)	71 (17%)	60 (18%)
**Outcomes**			
Sepsis on presentation based on pSOFA^1^	84 (25%)	99 (24%)	82 (25%)
Sepsis within 48 h based on pSOFA^1^	130 (39%)	161 (39%)	13 9 (42%)
Bacterial infection	104 (31%)	113 (27%)	95 (29%)
PICU admission	24 (7%)	26 (6%)	31 (9%)
Hospital length of stay (days)	1.16 (0.28, 3.08)	0.94 (0.23, 2.56)	1.58 (0.38, 3.07)
IPSCC criteria for sepsis met upon presentation	36 (11%)	40 (10%)	37 (11%)
IPSCC criteria for sepsis met within 48 h	57 (17%)	64 (15%)	64 (20%)

^1^ pSOFA, Pediatric Sequential Organ Failure Assessment Score; PICU, Pediatric Intensive Care Unit; IPSCC, International Pediatric Sepsis Consensus Conference.

### Primary outcome

**Figure 2:**
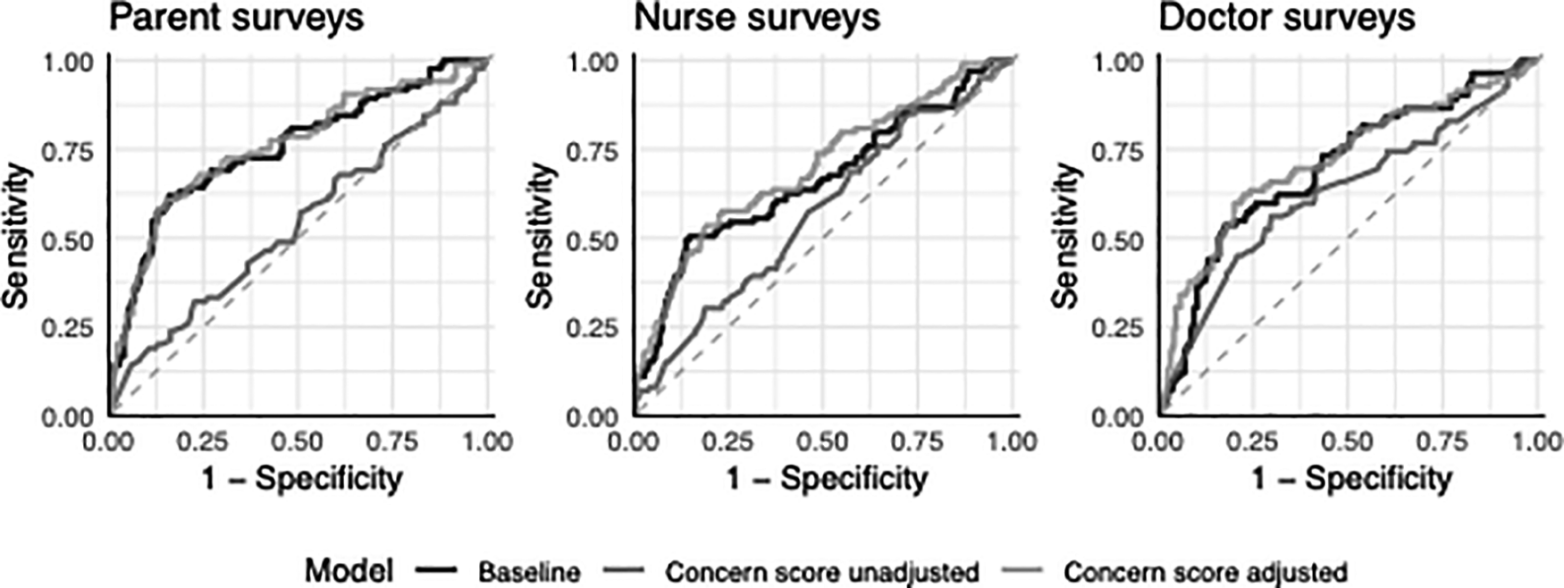
Diagnostic performance to predict sepsis on presentation for patients with parent, nurse, and doctor surveys.

The baseline model performed moderately well to predict sepsis on presentation (children with parent surveys: AUC 0.75; 95% CI: 0.69, 0.82) (Table 2). Parent concern was not associated with sepsis on presentation (AUC 0.53, 95% CI: 0.46, 0.61, aOR: 1.18; 95% CI: 0.89, 1.58), whereas nurse and doctor concern was significantly associated with sepsis (nurse: AUC 0.57, 95% CI: 0.50, 0.63, aOR: 1.43; 95% CI: 1.10, 1.87; doctor: AUC 0.63, 95% CI: 0.55, 0.70, aOR: 1.57; 95% CI: 1.20, 2.08). In the adjusted models, adding the concern score for the parent, nurse or doctor did not significantly improve the discriminative performance above the baseline model (Figure 2, Supplementary Material S9). Results using the best performing question (“Indicate how severe you think your child/patient's illness is today”) yielded similar performance to the concern score (Table 2).

**Table 2:** Odds ratios (ORs), 95% confidence intervals (CIs) and area under the receiver operator characteristic curve (AUC) to predict the primary outcome of sepsis at time of survey completion, with parents, nurses, and doctors concern as independent predictors. The columns each display the results for the children in relation to parent surveys, nurse surveys and doctor surveys, respectively. The baseline model is a multivariable model based on easily available observations at presentation. The concern score was derived from the exploratory factor analysis of responses to five questions assessing concern. The AUC and 95% confidence intervals (CI) of the AUC for each model are shown in italics.

Model	Parent		Nurse		Doctor	
	OR	95% CI	OR	95% CI	OR	95% CI
**Baseline Model**						
Heart rate	1.25	0.70, 2.23	1.16	0.70, 1.91	1.02	0.59, 1.77
Respiratory rate	1.15	0.54, 2.36	0.99	0.52, 1.83	0.92	0.45, 1.82
Age (months)	1.62	1.23, 2.14	1.39	1.09, 1.75	1.31	1.01, 1.70
Irritability	0.73	0.34, 1.49	0.88	0.47, 1.58	0.88	0.43, 1.73
Respiratory distress	1.36	0.57, 3.13	0.96	0.44, 1.97	1.30	0.62, 2.65
Chronic disease	5.18	2.75, 9.89	3.63	2.05, 6.45	3.77	2.03, 7.00
*AUC*	*0*.*75*	*0.69, 0.82*	*0*.*66*	*0.60, 0.73*	*0*.*70*	*0.63, 0.76*
**Unadjusted Models**						
Concern score	1.10	0.86, 1.42	1.29	1.02, 1.63	1.57	1.20, 2.08
*AUC*	*0*.*53*	*0.46, 0.61*	*0*.*57*	*0.50, 0.63*	*0*.*63*	*0.55, 0.70*
Best performing question^2^	1.14	0.89, 1.47	1.31	1.03, 1.67	1.53	1.16, 2.06
*AUC*	*0*.*55*	*0.48, 0.62*	*0*.*57*	*0.51, 0.63*	*0*.*61*	*0.54, 0.68*
**Adjusted Models** ^1^						
Concern score	1.18	0.89, 1.58	1.43	1.10, 1.87	1.61	1.21, 2.19
*AUC*	*0*.*76*	*0.70, 0.82*	*0*.*70*	*0.64, 0.76*	*0*.*72*	*0.65, 0.79*
Best performing question^2^	1.22	0.92, 1.63	1.40	1.08, 1.84	1.54	1.13, 2.12
*AUC*	*0*.*76*	*0.70, 0.83*	*0*.*70*	*0.64, 0.76*	*0*.*71*	*0.65, 0.78*

OR, Odds Ratio; CI, Confidence Interval; AUC, Area Under the receiver operating characteristic curve.

^1^ The variables in the baseline model were used as adjustment variables in the adjusted models.

^2^ Separate analyses were performed using the concern score and using the best performing question (“Indicate how severe you think your child/patient’s illness is today”) as the measure of concern.

### Secondary outcomes

In adjusted analyses, parent concern was not associated with the development of sepsis within 48 h of presentation (aOR: 1.28; 95% CI: 0.99, 1.67), while nurse (aOR: 1.68; 95% CI: 1.33, 2.14) and doctor (aOR: 1.78; 95% CI: 1.37, 2.33) concerns were (Supplementary Material S10). Parent concern was associated with confirmed or probable bacterial infection (aOR: 1.47; 95% CI: 1.14, 1.92), but not nurse (aOR: 1.07; 95% CI: 0.85, 1.34) or doctor concern (aOR: 1.12; 95% CI: 0.87, 1.44) (Supplementary Material S11). Concern was associated with longer hospital LOS when reported by the parent (aHR: 0.84; 95% CI: 0.75, 0.94), nurse (aHR: 0.78; 95% CI: 0.71, 0.86), and doctor (aHR: 0.75; 95% CI: 0.67, 0.84) (Supplementary Material S12). In unadjusted models, concerns reported by the parent (OR: 1.88; 95% CI: 1.17, 3.19), nurse (OR: 3.13; 95% CI: 1.91, 5.42) and doctor (OR: 2.97; 95% CI: 1.84, 5.15) were associated with PICU admission (Supplementary Material S13). In adjusted models, parent concern improved the predictive performance of the baseline model for bacterial infection only (difference in AUC 0.063; 95% CI: 0.004, 0.122). Both the nurse and doctor concerns significantly improved the performance of the baseline model for sepsis within 48 h and hospital LOS (Supplementary Material S9).

### Sensitivity analyses

When using the IPSCC criteria to define organ dysfunction, 43 (8.7%) children were diagnosed with sepsis on presentation and 76 (15.4%) within 48 h of presentation. Sensitivity analyses confirmed the main findings for both sepsis on presentation (Supplementary Material S14) and sepsis within 48 h of presentation (Supplementary Material S15).

## Discussion

This multicentre prospective observational cohort study assessed the diagnostic accuracy of parent and healthcare professional concern for illness severity in children evaluated for sepsis in the ED. As emphasised in the latest pediatric SSC, early recognition of pediatric sepsis remains of key importance (5), and delayed management contributes to poor patient outcomes. In our study, the parent was more likely than healthcare professionals to express a greater level of concern, irrespective of the severity of their child's illness. Parent concern was not associated with sepsis upon presentation or within 48 h, although it was associated with PICU admission, bacterial infection, and hospital LOS. Healthcare professional concern was associated with sepsis, and with sepsis within 48 h of presentation in both uni- and multivariate analyses. However, the inclusion of healthcare professional concern provided only a minor increment in diagnostic accuracy when compared to basic history and physiological variables available at presentation.

Clinical decision-making relies on the integration of a range of presumably objective diagnostic clues (derived from patient or chart assessment) resulting in a diagnosis, or a list of diagnostic possibilities (27). It has been postulated that experienced clinicians may recognise higher severity of illness through a subjective “gut feeling that something is wrong”, in addition to objective clinical signs and symptoms (28, 29). Parents can convey key information to the treating clinicians as they may assess disease severity and disease trajectories against their historical expertise on their child (“this disease is different from others”). In a study by Bruel et al., the greatest contextual factor which influenced a clinician's concern in addition to objective assessment, was parent concern (29). In this study conducted within the outpatient setting, parent concern significantly predicted the presence of severe infection (29). However, this single-center study did not adjust for severity, less than one percent of children had sepsis, and the authors do not report on organ dysfunction.

Contrary to our hypothesis, parent concern was not associated with the primary outcome of sepsis, which was defined as suspected infection with organ dysfunction and, among the three groups of respondents, was the least predictive of sepsis. This definition aligns with the current research and consensus among pediatric experts, who suggest that pediatric sepsis should be defined based on the presence of organ dysfunction, as indicated by a pSOFA score of >0 (5). This is due to the outdated nature of the current definition for pediatric sepsis, which relies on the Systemic Inflammatory Response Syndrome (SIRS) criteria from 2005 (21). The SIRS criteria have been deemed insufficiently specific and overly sensitive (21). Consequently, our primary outcome aligns with the evolving understanding of pediatric sepsis and the need for a more refined definition (20).

Sensitivity analyses using IPSCC criteria confirmed that concern provides little predictive value in predicting sepsis or the development of sepsis within 48hrs of presentation to the ED. Interestingly, parent concern was associated with an increased probability of bacterial infection, which is supported by findings of Urbane et al. (30). When adjusting for physiology and patient factors, parent concern did not contribute any additional value to these baseline factors in increasing the diagnostic accuracy of sepsis. Analyses resulted very similar when using the concern factor (which integrates information on all four questions), or when using the best performing question. Our results demonstrated that higher levels of concern were associated with subsequent admission to PICU. The surveys did not inquire specifically about sepsis, but rather about parental perceptions of illness severity in order to avoid reporting bias, as well as taking into account that parents may have limited knowledge of sepsis in general (31). The relationship between increased awareness and improved illness recognition has been well established (32, 33). In otitis media, for example, knowledge of acute otitis media and parent-reported symptoms have been identified as a tool to predict the illness more accurately (34). Determining the correlation between parent knowledge of sepsis and concern was outside the scope of this study. The children in our study were recruited at two large EDs, underwent active evaluation for sepsis, and severity adjustment was performed. It is possible that parent concern may have impacted which patients were screened for sepsis, resulting in possible enrolment bias. We acknowledge that contrary to a primary health setting, parents in our study had been concerned enough to bring their child to the ED, which may have impacted the performance of concern.

The findings that healthcare professional concern was associated with sepsis on presentation and the development of sepsis within 48 h of presentation were consistent with studies conducted by Bruel et al. and Oliva et al., that established that clinician concern substantially increased the risk of serious illness (29, 35). In the secondary outcomes, concern was associated with a greater likelihood of PICU admission and increased LOS. Sensitivity analyses based on IPSCC criteria confirmed the main results. Although our findings demonstrate that healthcare professional concern did slightly improve the diagnostic accuracy of sepsis within 48 h of presentation, the association between concern and the primary and other secondary outcomes did not lead to a substantial improvement in the prediction of concern when added to the baseline model. The baseline model consisted of measures indicating abnormal respiratory, cardiovascular, and neurologic status, in addition to age and comorbidity (36). Direct measures of organ dysfunction such as arterial hypotension were not included in the baseline model to avoid duplication with infection-associated organ dysfunction as the primary outcome. Due to sample size constraints, only these six variables were selected as measures of severity and indicators associated with sepsis. The overall predictive performance of the baseline model aligns with previous studies on sepsis recognition tools such as the Liverpool quick Sequential Organ Failure Assessment (LqSOFA), Pediatric Early Warning Score (PEWS), and National Institute for Health and Care Excellence (NICE) high-risk criteria (6, 7, 37).

Several limitations need to be considered. First, only 220 patients have a full set of parent, nurse and doctor surveys completed, which limited the ability to compare the association of concern for the same child across the three respondent groups. Furthermore, children may have been missed if the sepsis pathway was not used. A comparison of inter-rater reliability revealed substantial differences between the groups, indicating that the surveys were answered independently across the three respondent groups. Second, while standardised dissemination of study education was provided to local teams to reduce bias in delivery of information to parents and healthcare professionals completing the survey (Supplementary Material S16), all respondents were, by necessity of the clinical environment, aware of the child’s acute condition, history, and vital sign monitoring, none of which could be blinded. Third, although demographics of healthcare professionals were captured through title and years of experience, frequency of treating pediatric sepsis, details such as clinical sub-specialization, and training in sepsis were not captured and thus could not be modelled (38–40). Similarly, the survey measurement tool did not capture the demographics or socioeconomic status of the parent as well as any previous presentations to general practice. Fifth, as non-english speaking parents/caregivers were excluded, these population groups are under-represented in the findings. Sixth, while a substantial proportion of children had signs of organ dysfunction, the average acuity was low with only 7% of children requiring PICU admission. There were no fatalities. Finally, this study was conducted in a specialized pediatric ED setting in a high-income country, hence generalizability to other settings including mixed EDs, primary care, the ward or international settings may be limited. Moreover, the absence of an updated definition of pediatric sepsis is hinders the ability to conduct consistent and comparable research (20).

## Conclusion

While healthcare professional concern demonstrated some predictive value in improving recognition of sepsis in children, overall, the findings of this study do not support the use of parent or healthcare professional concern as a screening tool in isolation and rather we recommend concern be used in conjunction with physiologically relevant data. Parent concern, however, remains an important component of healthcare safety-netting. Parent concern may be more applicable to settings such as the community sector, and may be of value in the early identification of deterioration even in critical care settings (41, 42). Given that many national healthcare standards mandate the active involvement of families in decision making and care, this study helps to inform future approaches to assess parent concern (42). Increased education of healthcare professionals in sepsis may enhance potential benefits related to the inclusion of healthcare professional concern in the diagnostic process to recognize sepsis in a timely fashion.

## Data Availability

The raw data supporting the conclusions of this article will be made available by the authors, without undue reservation.
