# Supplementary material for: Impact of parental and healthcare professional concern on the diagnosis of pediatric sepsis: a diagnostic accuracy study
Source: Front Pediatr. 2023 Apr 17;11:1140121. doi: 10.3389/fped.2023.1140121 (PMC10149924; doi:10.3389/fped.2023.1140121)
Supplement: Supplementary file 1 [file Datasheet1.pdf]

**Supplementary materials for:**

*Impact of Parental and Healthcare Professional Concern on the Diagnosis of Pediatric Sepsis: A Diagnostic Accuracy Study*

*Zoe Sever[1] MScMed, Luregn J. Schlapbach[2,3,4] PhD, Patricia Gilholm[2] PhD, Melanie Jessup[1] PhD, Natalie Phillips[2,5] MD, Shane George [2,6,7] MD, Kristen Gibbons[2] PhD, Amanda Harley[1,2,6,8] MN on behalf of the Rapid Assessment of Paediatric Infectious Disease and Sepsis study investigators.**

**Corresponding Author:**

Zoe Sever

University of Queensland,

Brisbane 4101

QLD Australlia

zsev6807@uni.sydney.edu.au

**Supplementary Material Table of Contents:**

1. The Rapid Assessment of Paediatric Infectious Disease and Sepsis (RAPIDS) study investigators
2. Study surveys: a) parental, b) nursing and c) medical
3. Survey tool validation
4. Data classifications for types of infections and chronic diseases
5. Evaluation of unadjusted models of each concern question and the concern score for the primary outcome sepsis at time of survey completion
6. Development of baseline model
7. Demographics, clinical characteristics and primary and secondary outcomes for the total cohort, participants with all three surveys complete and participants with one or two surveys complete.
8. Proportion of responses for each concern question reported by the parent, nurse and doctor
9. Results for DeLong's test for two correlated ROC curves comparing the adjusted concern score model to the baseline model for relevant outcome and survey group. The difference in AUC and 95% confidence interval for the difference are also reported.
10. Odds ratios (OR), 95% confidence intervals (CI) and area under the receiver operator characteristic curve (AUC) for the models specified for the secondary outcome: Sepsis within 48 hours of presentation using pSOFA criteria. The columns display the results for the children with parent, nurse and doctor surveys, respectively.
11. Odds ratios (OR), 95% confidence intervals (CI) and area under the receiver operator characteristic curve (AUC) for the models specified for the secondary outcome: Bacterial infection. The columns display the results for the children with parent, nurse and doctor surveys, respectively.
12. Hazard ratios (HR), 95% confidence intervals (CI) and concordance statistics for the models specified for the secondary outcome: Hospital length of stay. The columns display the results for children with parent, nurse and doctor surveys, respectively
13. Odds ratios (OR), 95% confidence intervals (CI) and area under the receiver operator characteristic curve (AUC) for the models specified for the secondary outcome: PICU admission. The columns display the results for children with parent, nurse and doctor surveys, respectively.
14. Sensitivity analysis with the outcome infection with organ dysfunction, operationalized by IPSCC criteria. Odds ratios (OR), 95% confidence intervals (CI) and area under the receiver operator characteristic curve (AUC) are reported. The columns display the results for the children with parent, nurse and doctor surveys, respectively.
15. Odds ratios (OR), 95% confidence intervals (CI) and area under the receiver operator characteristic curve (AUC) for the models specified for the sensitivity outcome: IPSCC criteria within 48hrs of presentation. The columns display the results for children with parent, nurse and doctor surveys, respectively.
16. Standardised script for conducting surveys

**Supplementary Material 1: The Rapid Assessment of Paediatric Infectious Disease and Sepsis (RAPIDS) study investigators**

*Queensland Children's Hospital, Brisbane, Australia:* Luregn J Schlapbach, Sainath Raman, Natalie Phillips, Adam Irwin, Natalie Sharp, Melanie Kennedy, Amanda Harley, Roberta Ridolfi

*Child Health Research Center, The University of Queensland, Brisbane, Australia*: Zoe Sever, Kristen Gibbons, Trang Pham, Renate Le Marsney, Devika Ganesamoorthy

*Doherty Institute, University of Melbourne, Australia*: Lachlan Coin

*Gold Coast University Hospital, Southport, Australia*: Peter Snelling, Shane George, Keith Grimwood

*Diamantina Institue, The University of Queensland, Brisbane, Australia*: Antje Blumenthal

*Australian Centre for Ecogenomics, The University of Queensland, Brisbane, Australia:* Seweryn Bialasiewicz

*Townsville Base Hospital, Townsville, Australia*: Arjun Chavan, Luke Lawton, Eleanor Kitcatt

*Thursday Island Base Hospital, Thursday Island, Australia*: Allison Hempenstall

**Supplemental Material 2:**

**a) Parental Survey**

**Study ID:** …………………….

**Date &Time:** …………………….

| (Affix patient identification label here) |
|---|
| URN: |
| Family Name: |
| Given Names: |
| Address: |
| Date of Birth: Sex: ☐ M ☐ F ☐ I |

# Parent/Care-Giver

## RAPIDS Study

We would like to ask you to participate in this study. We are interested in measuring the parental concern as a tool to predict how severe a child's illness may be. No one knows your child better than you so we ask that you please complete this short survey to indicate if/what concerns you most about your child's illness. ***By completing this survey you are consenting to us analysing this data together with information from the medical health record for research purposes.***

**1. Please place an 'X' in the below fields to indicate your current level of concern regarding the severity of your child's illness:**

| Not Concerned | 1 | 2 | 3 | 4 | 5 | Extremely Concerned |
|---|---|---|---|---|---|---|
| | | | | | | |

**2. Do you have a gut feeling that something is wrong with your child? Please place an 'X' in the below fields to indicate your current level of concern regarding the severity of your child's illness:**

| I feel things are ok | 1 | 2 | 3 | 4 | 5 | I feel something is Extremely Wrong |
|---|---|---|---|---|---|---|
| | | | | | | |

**3. Please place an 'X' in the below fields to indicate how different is your child's behaviour right now compared to normal?**

| Normal Behaviour for Child | 1 | 2 | 3 | 4 | 5 | Extremely Abnormal Behaviour for Child |
|---|---|---|---|---|---|---|
| | | | | | | |

**4. What are the symptoms/behaviours that your child displayed, which prompted you to bring your child to hospital? *Please list as many as possible:***

1. ………………………………………………… 3. …………………………………………………

2. ………………………………………………… 4. …………………………………………………

**5. What was the symptom that concerned you the most?**

……………………………………………………………………………………………………………………

**6. Please place an 'X' in the below fields to indicate how severe you feel your child's illness is *today?***

| Mildly Unwell | 1 | 2 | 3 | 4 | 5 | Severely Unwell |
|---|---|---|---|---|---|---|
| | | | | | | |

**7. Please place an 'X' in the below fields to indicate how unwell you feel your child is today compared to *how unwell they have been in the past*:**

| Mild Illness for Child | 1 | 2 | 3 | 4 | 5 | Most Severe Illness for Child |
|---|---|---|---|---|---|---|
| | | | | | | |

b) **Nursing Survey**

**Study ID:** ……………………….

**Date &Time:** ……………………….

# Nursing Survey

## RAPIDS Trial

(Affix patient identification label here)

URN:

Family Name:

Given Names:

Address:

Date of Birth: Sex: ☐ M ☐ F ☐ I

We would like to ask you to participate in this study. We are interested in measuring the treating nurse's level of concern as a tool to predict how severe a child's illness may be. We ask that you please complete this short survey to indicate what concerns you most about your patient's illness. By completing this survey you are consenting to us including this data for research purposes.

**Job Title (RN/CN):** ……………………….

**Years of Experience:** ……………………….

**1. Please place an 'X' in the below fields to indicate your current level of concern regarding the severity of your patient's illness**

| Not Concerned | 1 | 2 | 3 | 4 | 5 | Extremely Concerned |
|---|---|---|---|---|---|---|
| | | | | | | |

**2. Do you have a gut feeling that something is wrong with your patient? Please place an 'X' in the below fields to indicate your current level of concern regarding the severity of your child's illness:**

| I feel things are ok | 1 | 2 | 3 | 4 | 5 | I feel something is Extremely Wrong |
|---|---|---|---|---|---|---|
| | | | | | | |

**3. Please place an 'X' in the below fields to indicate how different is your patient's behaviour deviating from their norm based on your discussion with patient/parent?**

| Normal Behaviour for Child | 1 | 2 | 3 | 4 | 5 | Extremely Abnormal Behaviour for Child |
|---|---|---|---|---|---|---|
| | | | | | | |

**4. What are the symptoms/behaviours that your patient displayed that trigger your concern? *Please list as many as possible***

**1.** ……………………………………………………… **3.** ………………………………………………………

**2.** ……………………………………………………… **4.** ………………………………………………………

**5. What was the symptom that concerned you the most?**

…………………………………………………………………………………………………………………………………

**6. Please place an 'X' in the below fields to indicate how severe you feel your patient's illness is:**

| Mildly Unwell | 1 | 2 | 3 | 4 | 5 | Severely Unwell |
|---|---|---|---|---|---|---|
| | | | | | | |

c) **Medical survey**

**Study ID:** ………………………….

**Date &Time:** ………………………….

| (Affix patient identification label here) |
|---|
| URN: |
| Family Name: |
| Given Names: |
| Address: |
| Date of Birth: Sex: ☐ M ☐ F ☐ I |

# Medical Survey

## RAPIDS Trial

We would like to ask you to participate in this study. We are interested in measuring the treating doctor's level of concern as a tool to predict how severe a child's illness may be. We ask that you please complete this short survey to indicate what concerns you most about your patient's illness. By completing this survey you are consenting to us including this data for research purposes.

**Job Title (e.g. SMO):** ………………………….

**Years of Experience:** ………………………….

**1. Please place an 'X' in the below fields to indicate your current level of concern regarding the severity of your patient's illness**

| Not Concerned | 1 | 2 | 3 | 4 | 5 | Extremely Concerned |
|---|---|---|---|---|---|---|
| | | | | | | |

**2. Do you have a gut feeling that something is wrong with your patient? Please place an 'X' in the below fields to indicate your current level of concern regarding the severity of your child's illness:**

| I feel things are ok | 1 | 2 | 3 | 4 | 5 | I feel something is Extremely Wrong |
|---|---|---|---|---|---|---|
| | | | | | | |

**3. Please place an 'X' in the below fields to indicate how different is your patient's behaviour deviating from their norm based on your discussion with patient/parent?**

| Normal Behaviour for Child | 1 | 2 | 3 | 4 | 5 | Extremely Abnormal Behaviour for Child |
|---|---|---|---|---|---|---|
| | | | | | | |

**4. What are the symptoms/behaviours that your patient displayed that trigger your concern?**
***<u>Please list as many as possible</u>***

1. …………………………………………………… 3. ……………………………………………………

2. …………………………………………………… 4. ……………………………………………………

**5. What was the symptom that concerned you the most?**

……………………………………………………………………………………………………………………

**6. Please place an 'X' in the below fields to indicate how severe you feel your patient's illness is:**

| Mildly Unwell | 1 | 2 | 3 | 4 | 5 | Severely Unwell |
|---|---|---|---|---|---|---|
| | | | | | | |

**Supplementary Material 3: Survey tool validation**

*Content Validity*

Four concern questions were applicable to all three survey types (parent, nurse, doctor). One concern question (Indicate how unwell you feel your child is today compared to how unwell they have been in the past) was only applicable to the parent. An exploratory factor analysis was conducted on the four concern questions included in the survey to identify if all four questions were measuring the same latent construct. A one-factor solution was estimated and compared to a two-factor solution. For the one-factor solution, the factor loadings for the four questions ranged from 0.68 to 0.89, indicating that each question was strongly correlated with one concern factor, and this factor explained 70% of the variation in concern ratings. Inclusion of a second factor showed that the additional factor explained only 2% of the variation in the responses, supporting a one-factor solution. A concern factor score was constructed using Bartlett`s least squares regression approachand was used as the composite score of concern in the regression analyses.(1)

*Internal consistency and inter-rater reliability*

Internal consistency was measured by Cronbach's alpha and strong internal consistency was observed for the responses from the parent, nurse and doctor (**Table 1**). The intra-class correlation was calculated to assess the inter-rater reliability between the parent, nurse and doctor for each concern question. A one-way analysis of variance, measuring the consistency between ratings was specified.(2) The intraclass correlations were low for all four concern questions indicating low agreement between ratings by the parent, nurse and doctor (**Table 1**).

**Table 1: Median and interquartile range for each question reported separately for the parent, nurse and doctor.** Chronbach's alpha for each response group and intra-class correlations (and 95% confidence intervals) for each question are also reported.

| Question | Parent Surveys | Nurse Surveys | Doctor Surveys | *Intra-Class Correlation (95%CI)* |
|---|---|---|---|---|
| **1. Indicate your current level of concern regarding the severity of your child/patient's illness.** | 4(3-4) | 3(2-4) | 3(2-4) | 0.20 (0.11, 0.29) |
| **2. Do you have a gut feeling that something is wrong with your child/patient** | 4(3-4) | 3(2-4) | 3(2-4) | 0.25 (0.17, 0.34) |
| **3. Indicate how different your child/patient is.** | 4(3-5) | 3(2-4) | 3(2-4) | 0.31 (0,23, 0.40) |
| **6. Indicate how severe you think your child/patient's illness is today.** | 4(3-4 | 3(2-4) | 3(2-4) | 0.26 (0.18, 0.35) |
| **7. Indicate how unwell you feel your child is today compared to how unwell they have been in the past.** | 4(3-5) | | | |
| ***Cronbach's alpha*** | 0.86 | 0.91 | 0.90 | |

**Supplementary Material 4: Data classifications for types of infections and chronic diseases**

| Data Question | Criteria |
|---|---|
| Was the main disease leading to presentation considered to be bacterial or viral? | 1. Definite Bacterial Infection - Sterile-site pathogenic bacteria that match syndrome<br>2. Probable Bacterial Infection and treated with antibiotics for at least 5 days - Bacterial syndrome but no bacteria identified and CRP >60mg/L<br>3. Unknown Bacterial or Viral Infection - Inconclusive or microbiology does not fit syndrome<br>4. Probable Viral Infection - Viral syndrome, but no virus identified<br>5. Definite Viral Infection - Virus identified that matches syndrome<br>6. Non-Bacterial, Non-Viral Infection or Non-Infectious Illness |
| Does the patient have any chronic diseases?<br><br>For example Congenital Malformation, Cystic Fibrosis, Chronic Lung Disease, Asthma, Congenital Health Defect, Chronic Renal Failure, Chronic Hepatic Insufficiency, Oncology Disease, Neuromuscular Disorder, Cerebral Palsy, Severe Encephalopathy, Metabolic Disease, Primary Immunodeficiency etc. | 1. Yes<br>2. No |

**Supplementary Material 5: Evaluation of unadjusted models of each concern question and the concern score for the primary outcome of sepsis at time of survey completion.**

Bivariate logistic regression models were performed to evaluate the unadjusted association between each of the concern questions and the concern score with the primary outcome, sepsis at time of survey completion. The models revealed no substantial differences in the effect size or the predictive performance between the four questions and the concern score. Therefore, only the concern score was reported for all analyses, along with the best performing question (Question 6: “Indicate how severe you think your child/patient’s illness is today”) as a comparison.

Table 2: Odds ratios and AUC (with 95% confidence intervals) for unadjusted models of the four concern questions and the concern score, predicting the primary outcome sepsis at time of survey completion.

| **Unadjusted Model** | **Parent** | | **Nurse** | | **Doctor** | |
|---|---|---|---|---|---|---|
| | OR | 95% CI | OR | 95% CI | OR | 95% CI |
| Question 1: Indicate your current level of concern regarding the severity of your child/patient’s illness. | 1.04 | 0.80, 1.37 | 1.18 | 0.94, 1.49 | 1.64 | 1.22, 2.23 |
| *AUC* | *0.52* | *0.44, 0.59* | *0.55* | *0.49, 0.61* | *0.61* | *0.54, 0.68* |
| Question 2: Do you have a gut feeling that something is wrong with your child/patient. | 1.09 | 0.86, 1.40 | 1.26 | 1.02, 1.57 | 1.55 | 1.19, 2.04 |
| *AUC* | *0.53* | *0.46, 0.60* | *0.57* | *0.51, 0.63* | *0.62* | *0.55, 0.69* |
| Question 3: Indicate how different your child/patient is. | 1.00 | 0.82, 1.24 | 1.24 | 1.01, 1.53 | 1.22 | 0.97, 1.55 |
| *AUC* | *0.51* | *0.44, 0.58* | *0.57* | *0.50, 0.63* | *0.61* | *0.54, 0.68* |
| Question 6[1]:Indicate how severe you think your child/patient’s illness is today. | 1.14 | 0.89, 1.47 | 1.31 | 1.03, 1.67 | 1.53 | 1.16, 2.06 |
| *AUC* | *0.55* | *0.48, 0.62* | *0.57* | *0.51, 0.63* | *0.61* | *0.54, 0.68* |
| Concern Score | 1.10 | 0.86, 1.42 | 1.29 | 1.02, 1.63 | 1.57 | 1.20, 2.08 |
| *AUC* | *0.53* | *0.46, 0.61* | *0.57* | *0.50, 0.63* | *0.63* | *0.55, 0.70* |

[1] Question 6 is used in all models to compare to the concern score.
OR odds ratio, CI Confidence interval, AUC area under the receiver operating characteristic curve

**Supplementary Material 6: Development of Baseline Model**

The baseline model was constructed using physiological and patient characteristics available upon presentation. In order to avoid overfitting the regression models, and to ensure the same baseline covariates were used for all outcomes, six variables were selected a priori as measures of severity associated with sepsis.(3) These variables were age in months, elevated heart rate and elevated respiratory rate on presentation, defined as heart rate/respiratory rate greater than the 90th centile for a child's age (4), irritability and respiratory distress at time of presentation, and chronic disease. For outcomes with less than 70 events (corresponding to less than 10 events-per-variable), only the unadjusted models were estimated, as adjustment for the baseline model would result in overfitting.

**Supplementary Material 7: Demographics, clinical characteristics and primary and secondary outcomes for the total cohort, participants with all three surveys complete and participants with one or two surveys complete.**

| **Characteristic** | **Total Cohort**, N = 492 | **Participants with all three surveys completed**, N = 220 | **Participants with one or two surveys**, N = 272 |
|---|---|---|---|
| **Demographics** | | | |
| Age (months)[1] | 26.8 (13.2, 70.7) | 29.5 (13.7, 77.8) | 21.6 (13.1, 59.2) |
| Male | 268 (54%) | 130 (59%) | 138 (51%) |
| Weight (kg)[1] | 13.3 (10.4, 21.8) | 13.5 (10.4, 24.0) | 13.0 (10.4, 20.2) |
| Unknown | 9 | 5 | 4 |
| **Symptoms at presentation** | | | |
| Fever | 428 (87%) | 184 (84%) | 244 (90%) |
| Rash | 63 (13%) | 34 (15%) | 29 (11%) |
| Altered level of consciousness | 55 (11%) | 29 (13%) | 26 (10%) |
| Irritability | 114 (23%) | 54 (25%) | 60 (22%) |
| Pain | 144 (29%) | 65 (30%) | 79 (29%) |
| Nausea/vomiting | 139 (28%) | 61 (28%) | 78 (29%) |
| Respiratory distress/Apnoea | 72 (15%) | 36 (16%) | 36 (13%) |
| Cough | 146 (30%) | 68 (31%) | 78 (29%) |
| Other symptoms | 226 (46%) | 109 (50%) | 117 (43%) |
| Clinical focus of infection present?[3] | 171 (35%) | 82 (37%) | 89 (33%) |
| Respiratory | 96 (56%) | 41 (50%) | 55 (62%) |
| Renal | 17 (10%) | 7 (9%) | 10 (11%) |
| Nervous | 3 (2%) | 1 (1%) | 2 (2%) |
| Cardiovascular | 7 (4%) | 4 (5%) | 3 (3%) |
| Immune | 9 (5%) | 7 (9%) | 2 (2%) |
| Musculoskeletal | 19 (11%) | 10 (12%) | 9 (10%) |
| Integumentary | 1 (1%) | 1 (1%) | 0 (0%) |
| Oral | 3 (2%) | 1 (1%) | 2 (2%) |
| Gastrointestinal | 16 (9%) | 10 (12%) | 6 (7%) |
| Other | 0 (0%) | 0 (0%) | 0 (0%) |
| **Infectious risk assessment** | | | |
| Ex-prematurity (< 37 weeks) | 27 (6%) | 13 (6%) | 14 (5%) |
| Has the patient had major surgery or significant burns in the last 14 days? | 14 (3%) | 7 (3%) | 7 (3%) |
| Does the patient have chronic disease? | 81 (16%) | 42 (19%) | 39 (14%) |
| Congenital Malformation | 10 (2%) | 5 (2%) | 5 (2%) |
| Chronic Lung Disease | 4 (1%) | 1 (0.4%) | 3 (1%) |
| Asthma | 11 (2%) | 5 (2%) | 6 (2%) |
| Congenital Heart Defect | 3 (1%) | 2 (1%) | 1 (0.4%) |
| Chronic Renal Failure | 3 (1%) | 2 (1%) | 1 (0.4%) |
| Oncology Disease | 21 (4%) | 13 (6%) | 8 (3%) |
| Neuromuscular Disorder | 2 (0.4%) | 1 (0.4%) | 1 (0.4%) |
| Cerebral Palsy, Severe Encephalopathy | 17 (3%) | 7 (3%) | 10 (4%) |
| Metabolic Disease | 1 (0.2%) | 0 (0%) | 1 (0.4%) |
| Other major chronic disease | 31 (6%) | 16 (7%) | 15 (5%) |
| **Severity on presentation** | | | |
| Heart rate[2] | 140.4 (30.5) | 136.9 (31.8) | 143.3 (29.2) |
| Unknown | 2 | 2 | 0 |
| Respiratory rate[2] | 34.1 (11.6) | 33.7 (12.5) | 34.4 (10.7) |
| Systolic blood pressure[2] | 108.2 (14.9) | 106.9 (15.3) | 109.1 (14.7) |
| Unknown | 144 | 71 | 73 |

| | | | |
|---|---|---|---|
| Glasgow coma scale[1] | 15.0 (15.0, 15.0) | 15.0 (15.0, 15.0) | 15.0 (15.0, 15.0) |
| Unknown | 9 | 3 | 6 |
| Capillary refill (seconds) | | | |
| < 2 seconds | 35 (7%) | 16 (7%) | 19 (7%) |
| 2-5 seconds | 445 (93%) | 201 (93%) | 244 (93%) |
| >5 seconds | 0 (0%) | 0 (0%) | 0 (0%) |
| Not measured | 12 (3%) | 3 (1%) | 9 (4%) |
| C-reactive protein[1] | 21.0 (5.1, 68.0) | 20.0 (3.9, 65.0) | 23.0 (6.2, 69.2) |
| Unknown | 167 | 59 | 108 |
| White cell count[1] | 11.2 (7.3, 15.8) | 10.5 (6.8, 15.5) | 11.8 (8.0, 16.0) |
| Unknown | 133 | 38 | 95 |
| Lactate[1] | 1.7 (1.2, 2.6) | 1.8 (1.2, 2.7) | 1.7 (1.2, 2.4) |
| Unknown | 250 | 99 | 151 |
| **Other** | | | |
| Invasive ventilation | 8 (2%) | 7 (3%) | 1 (0.4%) |
| Non-invasive ventilation | 6 (1%) | 2 (1%) | 4 (2%) |
| Inotropes | 2 (0.4%) | 2 (1%) | 0 (0%) |
| **Primary Outcome** | | | |
| Sepsis at time of survey – pSOFA criteria[4] | 118 (24%) | 55 (25%) | 63 (23%) |
| **Sensitivity Outcomes** | | | |
| Sepsis at time of survey – IPSCC criteria[4] | 43 (9%) | 28 (13%) | 15 (6%) |
| Sepsis within 48 hours of presentation – IPSCC criteria[4] | 76 (15%) | 43 (20%) | 33 (12%) |
| **Secondary Outcomes** | | | |
| Sepsis within 48 hours of presentation – pSOFA criteria[4] | 191 (39%) | 94 (43%) | 97 (36%) |
| PICU admission[4] | 31 (6%) | 21 (10%) | 10 (4%) |
| Confirmed or probable bacterial infection[4] | 133 (27%) | 69 (31%) | 64 (24%) |
| Hospital length of stay[1,4] | 1.0 (0.2, 2.8) | 1.3 (0.3, 3.2) | 0.8 (0.2, 2.2) |
| Was the main diagnosis infection related? | 434 (88%) | 201 (91%) | 233 (86%) |
| Was the main disease leading to presentation considered to be bacterial or viral? | | | |
| Definite Bacterial Infection | 72 (15%) | 40 (18%) | 32 (12%) |
| Probable Bacterial Infection | 61 (12%) | 29 (13%) | 32 (12%) |
| Unknown Bacterial or Viral Infection | 95 (19%) | 42 (19%) | 53 (19%) |
| Probable Viral Infection | 171 (35%) | 66 (30%) | 105 (39%) |
| Definite Viral Infection | 69 (14%) | 33 (15%) | 36 (13%) |
| Non-Bacterial, Non-Viral Infection or Non-Infectious Illness | 24 (5%) | 10 (4%) | 14 (5%) |

[1]Median (IQR); [2]Mean (SD); [3] Percentages displayed for each source of infection are of the total number with a clinical focus of infection present; [4] Primary, secondary and sensitivity outcomes used for the analyses

IPSCC: International Pediatric Sepsis Consensus Conference, pSOFA: Pediatric Sequential Organ Failure Assessment

**Supplementary Material 8: Proportion of responses for each concern question reported by the parent, nurse and doctor.**

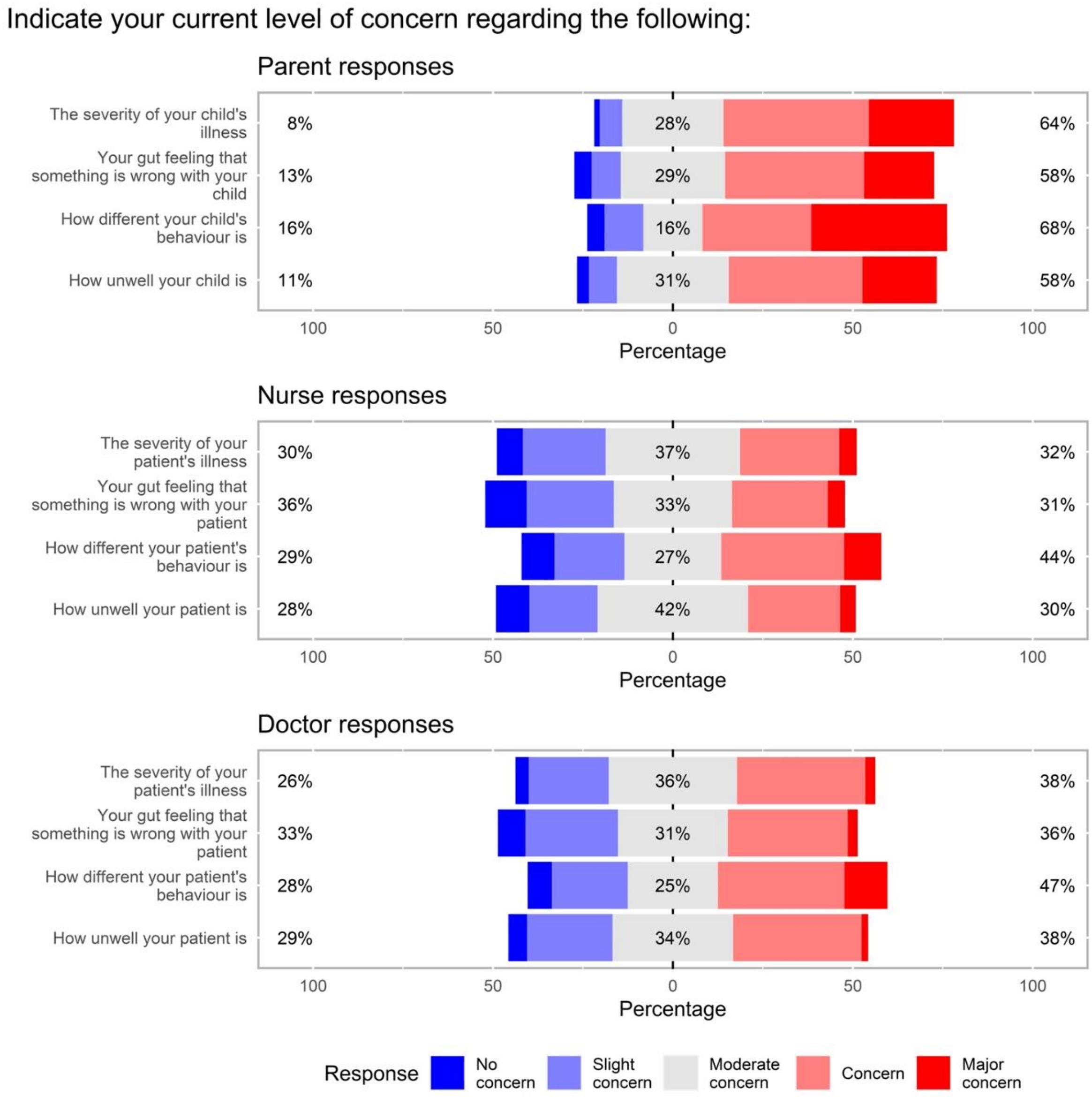

**Supplementary Material 9: Results for DeLong's test for two correlated ROC curves comparing the adjusted concern score model to the baseline model for relevant outcome and survey group.** The difference in AUC and 95% confidence intervals (CIs) for the difference are also reported.*

| Outcome | Z | AUC baseline model | AUC adjusted model | Difference in AUC | 95% CI of difference |
|---|---|---|---|---|---|
| **Parent surveys** | | | | | |
| Sepsis at time of presentation | 0.63 | 0.751 | 0.756 | 0.005 | -0.010, 0.019 |
| Sepsis within 48 hours | 1.33 | 0.740 | 0.751 | 0.011 | -0.005, 0.027 |
| Bacterial infection | 2.10 | 0.566 | 0.629 | 0.063 | 0.004, 0.122 |
| Hospital LOS[1] | 1.89 | 0.608 | 0.631 | 0.023 | -0.001, 0.047 |
| **Nurse surveys** | | | | | |
| Sepsis at time of presentation | 1.63 | 0.663 | 0.697 | 0.034 | -0.007, 0.074 |
| Sepsis within 48 hours | 2.39 | 0.674 | 0.719 | 0.045 | 0.008, 0.082 |
| Bacterial infection | -0.39 | 0.589 | 0.585 | -0.004 | -0.022, 0.015 |
| Hospital LOS[1] | 4.17 | 0.578 | 0.634 | 0.056 | 0.030, 0.082 |
| **Doctor surveys** | | | | | |
| Sepsis at time of presentation | 0.75 | 0.697 | 0.716 | 0.020 | -0.032, 0.071 |
| Sepsis within 48 hours | 2.01 | 0.697 | 0.740 | 0.043 | 0.001, 0.084 |
| Bacterial infection | -0.03 | 0.585 | 0.585 | -0.001 | -0.031, 0.030 |
| Hospital LOS[1] | 4.22 | 0.584 | 0.657 | 0.073 | 0.039, 0.107 |

[1] For hospital LOS, the difference in concordance was calculated.

*PICU admission and the sepsis outcomes defined by the IPSCC criteria are not included as only unadjusted models were performed for these outcomes.

IPSCC: International Pediatric Sepsis Consensus Conference, CI: Confidence Interval, AUC: Area Under the receiver operating characteristic Curve, PICU: Pediatric Intensive Care Unit, LOS: Length of Stay

**Supplementary Material 10: Odds ratios (OR), 95% confidence intervals (CI) and area under the receiver operator characteristic curve (AUC) for the models specified for the secondary outcome: Sepsis within 48 hours of presentation using pSOFA criteria.** The columns display the results for the children with parent, nurse and doctor surveys, respectively.

| Model | Parent | | Nurse | | Doctor | |
|---|---|---|---|---|---|---|
| | **OR** | **95% CI** | **OR** | **95% CI** | **OR** | **95% CI** |
| **Baseline Model** | | | | | | |
| Heart rate | 1.58 | 0.95, 2.65 | 1.47 | 0.95, 2.28 | 1.35 | 0.83, 2.19 |
| Respiratory rate | 1.16 | 0.60, 2.20 | 0.88 | 0.51, 1.50 | 1.04 | 0.56, 1.91 |
| Age (months) | 1.59 | 1.22, 2.09 | 1.37 | 1.10, 1.72 | 1.32 | 1.03, 1.70 |
| Irritability | 0.66 | 0.35, 1.22 | 0.79 | 0.47, 1.31 | 0.77 | 0.43, 1.38 |
| Respiratory distress | 2.40 | 1.13, 5.15 | 1.86 | 1.00, 3.46 | 2.00 | 1.05, 3.86 |
| Chronic disease | 5.05 | 2.61, 10.18 | 3.45 | 1.96, 6.23 | 4.03 | 2.14, 7.90 |
| *AUC* | *0.74* | *0.68, 0.80* | *0.67* | *0.62, 0.73* | *0.70* | *0.64, 0.75* |
| **Unadjusted Models** | | | | | | |
| Concern score | 1.24 | 0.99, 1.56 | 1.56 | 1.27, 1.94 | 1.78 | 1.40, 2.28 |
| *AUC* | *0.56* | *0.50, 0.63* | *0.62* | *0.57, 0.68* | *0.65* | *0.59, 0.71* |
| Question 6 | 1.27 | 1.01, 1.60 | 1.55 | 1.25, 1.93 | 1.80 | 1.40, 2.35 |
| *AUC* | *0.57* | *0.51, 0.63* | *0.62* | *0.57,0.67* | *0.65* | *0.59, 0.70* |
| **Adjusted Models** | | | | | | |
| Concern score | 1.28 | 0.99, 1.67 | 1.68 | 1.33, 2.14 | 1.78 | 1.37, 2.33 |
| *AUC* | *0.75* | *0.70, 0.81* | *0.72* | *0.67, 0.77* | *0.74* | *0.69, 0.80* |
| Question 6 | 1.31 | 1.01, 1.71 | 1.60 | 1.27, 2.04 | 1.77 | 1.34, 2.36 |
| *AUC* | *0.75* | *0.70, 0.81* | *0.72* | *0.67, 0.77* | *0.73* | *0.68, 0.79* |

OR: Odds Ratio, CI: Confidence Interval, AUC: Area Under the receiver operating characteristic curve

**Supplementary Material 11: Odds ratios (OR), 95% confidence intervals (CI) and area under the receiver operator characteristic curve (AUC) for the models specified for the secondary outcome: Bacterial infection.** The columns display the results for the children with parent, nurse and doctor surveys, respectively.

| Model | Parent | | Nurse | | Doctor | |
|---|---|---|---|---|---|---|
| | **OR** | **95% CI** | **OR** | **95% CI** | **OR** | **95% CI** |
| **Baseline Model** | | | | | | |
| Heart rate | 1.22 | 0.75, 1.98 | 0.93 | 0.59, 1.46 | 1.13 | 0.68, 1.85 |
| Respiratory rate | 1.12 | 0.59, 2.08 | 0.80 | 0.43, 1.41 | 0.81 | 0.42, 1.52 |
| Age (months) | 1.21 | 0.94, 1.55 | 1.25 | 0.99, 1.56 | 1.30 | 1.01, 1.66 |
| Irritability | 0.87 | 0.48, 1.54 | 0.87 | 0.50, 1.47 | 0.92 | 0.49, 1.67 |
| Respiratory distress | 0.91 | 0.41, 1.90 | 1.06 | 0.53, 2.05 | 1.00 | 0.49, 1.97 |
| Chronic disease | 0.96 | 0.50, 1.79 | 0.87 | 0.46, 1.57 | 0.90 | 0.46, 1.69 |
| *AUC* | *0.57* | *0.50, 0.63* | *0.59* | *0.53, 0.65* | *0.59* | *0.52, 0.65* |
| **Unadjusted Models** | | | | | | |
| Concern score | 1.47 | 1.15, 1.90 | 1.01 | 0.82, 1.26 | 1.08 | 0.85, 1.38 |
| *AUC* | *0.60* | *0.53, 0.66* | *0.50* | *0.44, 0.56* | *0.52* | *0.45, 0.59* |
| Question 6 | 1.46 | 1.14, 1.89 | 1.02 | 0.82, 1.27 | 1.07 | 0.83, 1.38 |
| *AUC* | *0.59* | *0.53, 0.66* | *0.50* | *0.44, 0.56* | *0.52* | *0.46, 0.59* |
| **Adjusted Models** | | | | | | |
| Concern score | 1.47 | 1.14, 1.92 | 1.07 | 0.85, 1.34 | 1.12 | 0.87, 1.44 |
| *AUC* | *0.63* | *0.57, 0.70* | *0.59* | *0.53, 0.65* | *0.59* | *0.52, 0.65* |
| Question 6 | 1.46 | 1.13, 1.90 | 1.07 | 0.85, 1.34 | 1.11 | 0.85, 1.46 |
| *AUC* | *0.63* | *0.56, 0.69* | *0.58* | *0.52, 0.64* | *0.59* | *0.52, 0.65* |

OR: Odds Ratio; CI: Confidence Interval, AUC: Area Under the receiver operating characteristic Curve

**Supplementary Material 12: Hazard ratios (HR), 95% confidence intervals (CI) and concordance statistics for the models specified for the secondary outcome: Hospital length of stay.** The columns display the results for children with parent, nurse and doctor surveys, respectively

| **Model** | **Parent** | | **Nurse** | | **Doctor** | |
|---|---|---|---|---|---|---|
| | **HR** | **95% CI** | **HR** | **95% CI** | **HR** | **95% CI** |
| **Baseline Model** | | | | | | |
| Heart rate | 0.91 | 0.72, 1.15 | 0.94 | 0.77, 1.15 | 1.04 | 0.82, 1.31 |
| Respiratory rate | 0.99 | 0.72, 1.35 | 1.17 | 0.90, 1.53 | 0.88 | 0.65, 1.21 |
| Age (months) | 0.96 | 0.86, 1.07 | 0.96 | 0.87, 1.06 | 0.97 | 0.87, 1.09 |
| Irritability | 1.18 | 0.91, 1.54 | 1.10 | 0.87, 1.39 | 1.10 | 0.84, 1.44 |
| Respiratory distress | 0.69 | 0.48, 0.99 | 0.68 | 0.50, 0.92 | 0.72 | 0.52, 1.00 |
| Chronic disease | 0.67 | 0.50, 0.91 | 0.68 | 0.52, 0.90 | 0.71 | 0.53, 0.95 |
| *Concordance* | *0.61* | *0.57, 0.64* | *0.58* | *0.55, 0.61* | *0.58* | *0.55, 0.62* |
| **Unadjusted Models** | | | | | | |
| Concern score | 0.88 | 0.80, 0.98 | 0.80 | 0.73, 0.88 | 0.74 | 0.66, 0.82 |
| *Concordance* | *0.57* | *0.53, 0.61* | *0.61* | *0.58, 0.64* | *0.63* | *0.60, 0.67* |
| Question 6 | 0.92 | 0.84, 1.02 | 0.75 | 0.69, 0.83 | 0.71 | 0.63, 0.80 |
| *Concordance* | *0.56* | *0.52, 0.59* | *0.62* | *0.59, 0.65* | *0.63* | *0.60, 0.66* |
| **Adjusted Models** | | | | | | |
| Concern score | 0.84 | 0.75, 0.94 | 0.78 | 0.71, 0.86 | 0.75 | 0.67, 0.84 |
| *Concordance* | *0.63* | *0.60, 0.66* | *0.63* | *0.60, 0.67* | *0.66* | *0.62, 0.69* |
| Question 6 | 0.89 | 0.80, 0.99 | 0.75 | 0.68, 0.82 | 0.73 | 0.65, 0.82 |
| *Concordance* | *0.63* | *0.59, 0.66* | *0.65* | *0.62, 0.68* | *0.66* | *0.62, 0.69* |

HR: Hazard Ratio; CI: Confidence Interval, AUC: Area Under the receiver operating characteristic Curve

**Supplementary Material 13: Odds ratios (OR), 95% confidence intervals (CI) and area under the receiver operator characteristic curve (AUC) for the models specified for the secondary outcome: PICU admission.** The columns display the results for children with parent, nurse and doctor surveys, respectively.*

| **Model** | **Parent** | | **Nurse** | | **Doctor** | |
|---|---|---|---|---|---|---|
| | **OR** | **95% CI** | **OR** | **95% CI** | **OR** | **95% CI** |
| **Unadjusted Models** | | | | | | |
| Concern Factor | 1.88 | 1.17, 3.19 | 3.13 | 1.91, 5.42 | 2.97 | 1.84, 5.15 |
| *AUC* | *0.65* | *0.53, 0.77* | *0.77* | *0.68, 0.87* | *0.75* | *0.66, 0.84* |
| Question 6: "Indicate how severe you think your child/patient's illness is today" | 1.54 | 0.99, 2.51 | 3.00 | 1.85, 5.09 | 4.10 | 2.31, 8.12 |
| *AUC* | *0.62* | *0.49, 0.74* | *0.75* | *0.66, 0.84* | *0.76* | *0.68, 0.84* |

OR: Odds Ratio; CI: Confidence Interval, AUC: Area Under the receiver operating characteristic curve

* Adjusted models were not performed due to low prevalence of the outcome.

**Supplementary Material 14: Sensitivity analysis with the outcome infection with organ dysfunction, operationalized by IPSCC criteria.** Odds ratios (OR), 95% confidence intervals (CI) and area under the receiver operator characteristic curve (AUC) are reported. The columns display the results for the children with parent, nurse and doctor surveys, respectively.*

| Model | Parent | | Nurse | | Doctor | |
|---|---|---|---|---|---|---|
| | **OR** | **95% CI** | **OR** | **95% CI** | **OR** | **95% CI** |
| **Unadjusted Models** | | | | | | |
| Concern score | 1.20 | 0.84, 1.74 | 1.78 | 1.25, 2.60 | 1.47 | 1.02, 2.17 |
| *AUC* | *0.56* | *0.45, 0.67* | *0.64* | *0.54, 0.74* | *0.60* | *0.50, 0.70* |
| Question 6 | 1.21 | 0.85, 1.76 | 1.90 | 1.32, 2.79 | 1.47 | 1.00, 2.23 |
| *AUC* | *0.57* | *0.47, 0.67* | *0.66* | *0.58, 0.74* | *0.60* | *0.50, 0.70* |

OR: Odds Ratio; CI: Confidence Interval, AUC: Area Under the receiver operating characteristic Curve, IPSCC: International Pediatric Sepsis Consensus Conference

* Adjusted models were not performed due to low prevalence of the outcome.

**Supplementary Material 15: Odds ratios (OR), 95% confidence intervals (CI) and area under the receiver operator characteristic curve (AUC) for the models specified for the sensitivity outcome: IPSCC criteria within 48hrs of presentation.** The columns display the results for children with parent, nurse and doctor surveys, respectively.*

| Model | Parent | | Nurse | | Doctor | |
|---|---|---|---|---|---|---|
| | **OR** | **95% CI** | **OR** | **95% CI** | **OR** | **95% CI** |
| **Unadjusted Models** | | | | | | |
| Concern score | 1.10 | 0.82, 1.48 | 1.74 | 1.30, 2.36 | 1.68 | 1.21, 2.23 |
| *AUC* | *0.53* | *0.44, 0.62* | *0.64* | *0.57, 0.72* | *0.63* | *0.56, 0.70* |
| Question 6 | 1.15 | 0.86, 1.56 | 1.70 | 1.27, 2.32 | 1.86 | 1.34, 2.64 |
| *AUC* | *0.56* | *0.48, 0.64* | *0.64* | *0.57, 0.71* | *0.64* | *0.58, 0.71* |

OR: Odds Ratio; CI: Confidence Interval, AUC: Area Under the receiver operating characteristic curve, IPSCC: International Pediatric Sepsis Consensus Conference

* Adjusted models were not performed due to low prevalence of the outcome.

## Supplementary Material 16: standardised script for conducting surveys

In order to reduce unintended bias when conducting concern surveys with parents, the following standardized script has been developed.

Mentioning certain trigger words such as "sepsis" "septic pathway" "organ dysfunction" or "death" could possibly increase parental concern or anxiety and skew responses. If possible, we want to minimise this bias.

The survey should *ideally* be distributed at time of screening for sepsis via the sepsis pathway.

1. **Introduce yourself**

*Hello, my name is ___. I am a Registered Nurse and part of the Pediatric Research Team here in Emergency.*

2. **State the research being conducted:**

*Currently in the department, we are conducting research investigating parental concern levels as a tool to predict illness in children.*

*What this means is that we want to find out how your concern levels as a parent can improve recognition of illness.*

3. **Ask consent for survey**

*This would involve you completing a short 1 minute survey. If you like I can read out the questions and go through it with you. We will also be completing surveys assessing concern levels of the nurse and doctor.*

4. **Complete questionnaire**

Throughout the survey, read questions exactly as written on questionnaire, and do not change the wording. Go through the questions in order.

Avoid asking leading questions. See an example in red:
**Researcher**: What are the symptoms or behaviours that your child displayed, which prompted you to come to hospital? Was it the fever and shortness of breath?

In terms of handing out parent / carer information leaflets – this should take place ideally after survey has been completed. Researchers should also encourage the bedside nurse to educate further where necessary.

5. **Ask parent if they have any questions relating to the study**

6. **Thank parent for their participation**

**References:**


1. Grice JW. Computing and evaluating factor scores. Psychol Methods. 2001;6(3):430-50.
2. Shrout PE, Fleiss JL. Intraclass correlations: uses in assessing rater reliability. Psychol Bull. 1979;86(2):420-8.
3. Menon K, Schlapbach LJ, Akech S, Argent A, Biban P, Carrol ED, et al. Criteria for Pediatric Sepsis—A Systematic Review and Meta-Analysis by the Pediatric Sepsis Definition Taskforce. Critical Care Medicine. 2021.
4. Bonafide CP, Brady PW, Keren R, Conway PH, Marsolo K, Daymont C. Development of heart and respiratory rate percentile curves for hospitalized children. Pediatrics (Evanston). 2013;131(4):e1150-e7.
